# Batch Fabrication of Broadband Metallic Planar Microlenses and Their Arrays Combining Nanosphere Self-Assembly with Conventional Photolithography

**DOI:** 10.1186/s11671-017-2158-x

**Published:** 2017-06-02

**Authors:** Ping Wang, Xiaochang Yu, Yechuan Zhu, Yiting Yu, Weizheng Yuan

**Affiliations:** 1 Key Laboratory of Micro/Nano Systems for Aerospace, Ministry of Education, Northwestern Polytechnical University, Xi’an, 710072 China; 2 Shaanxi Province Key Laboratory of Micro and Nano Electro-Mechanical Systems, Northwestern Polytechnical University, Xi’an, 710072 China

**Keywords:** Planar microlens, Batch fabrication, Nanosphere lithography, Self-assembly

## Abstract

A novel low-cost, batch-fabrication method combining the spin-coating nanosphere lithography (NSL) with the conventional photolithographic technique is demonstrated to efficiently produce the metallic planar microlenses and their arrays. The developed microlenses are composed of subwavelength nanoholes and can focus light effectively in the entire visible spectrum, with the foci sizes close to the Rayleigh diffraction limit. By changing the spacing and diameter of nanoholes, the focusing efficiency can be tuned. Although the random defects commonly exist during the self-assembly of nanospheres, the main focusing performance, e.g., focal length, depth of focus (DOF), and full-width at half-maximum (FWHM), keeps almost invariable. This research provides a cheap way to realize the integrated nanophotonic devices on the wafer level.

## Background

Microlenses as a class of most ubiquitous optical components, aiming to manipulate and focus light at the micro-/nanoscale, have important applications, such as display technology [1], laser beam collimation [2], molecular detection [3], and optical information storage [4]. Though refractive microlenses are extensively used in commercial devices with high optical throughput, they inevitably suffer from bulky size, chromatic, and spherical aberrations [5]. On the other hand, diffractive microlenses exhibit less aberration, but their physical size and complex three-dimensional (3D) surface profiles make them less useful in miniaturized and highly scaled devices. Furthermore, their fabrication requires precise alignment during multiple lithographic processes, which also limits their adoption in highly integrated micro-/nano-optical devices [6, 7].

Substantial efforts have been devoted to exploring plasmonics in recent years [8–10], due to the unique capability to route and manipulate light at the nanometer length scale. As an important category of plasmonic devices, plasmonic lenses based on thin nanostructured metallic films were proposed and developed [11–17]. Surface plasmons (SPs) on metallic films are excited by the interaction of incident light with the charge oscillations on the lens’ entrance surface and are squeezed into the nanoapertures. After passing through the whole metallic films in specific waveguide modes, SPs change to the propagating waves again. The sub-waves transmitting from all the nanoapertures will interfere with each other and form a light spot with the maximum intensity at a certain distance away from the lens’ exit surface, which are also named as the focusing spot and the focal plane. Consequently, metallic planar microlenses comprising of nanoaperture arrays are potential candidates for conventional dielectric-based refractive lenses, bringing out subwavelength, yet broadband focusing and allowing all-optical or opto-electronic single-chip integration. However, all the microlenses composed of nanostructures require the high-precision nanofabrication techniques, such as electron-beam lithography (EBL) and focused ion beam (FIB) milling. Although they are powerful tools for prototyping microlenses, these processes are expensive, time consuming, and not suitable for the large-area parallel fabrication.

Recently, a kind of microlens based on nanoholes capable of focusing all wavelengths in the visible spectrum to a single spot was reported by employing a batch-fabrication method of soft interference lithography (SIL) followed by a nanopatterning procedure [18]. Unfortunately, this method is not ideal for microlenses because the nanoholes around the periphery show significantly smaller diameter than that of the central ones, and some are even blocked, causing a large deviation of the focal length from the design. Therefore, developing a versatile and large-area fabrication technique for microlenses is crucial for their practical applications; nonetheless, the effective method using the current top-down or bottom-up approaches still remains a big challenge. Moreover, it is worthwhile to investigate the random defects on the focusing performance and the coupling effect between adjacent microlenses.

The promising large-area fabrication methods, such as photolithography, laser interference lithography (LIL), and nanosphere lithography (NSL), enable the creation of various nanostructures. Photolithography is widely used in microelectronics to manufacture integrated circuits (ICs). The combination of short-wavelength light sources, including deep ultraviolet (DUV) and extreme ultraviolet (EUV), and innovations, such as immersion lithography and phase shift masks, have pushed the feature size well into the nanometer scale [19, 20]. Although the traditional mask-based optical lithography is well established and widely used in the IC industry, it is also very expensive both to set up and to operate. As a much simpler and cheaper scale methodology, LIL is based on the interference of several coherent laser beams and can produce one-dimensional (1D), two-dimensional (2D), and 3D periodic structures with feature dimensions approaching 20 nm [21]. But suffering from the restriction of technology, LIL is difficult to produce the patterns over centimeter scale [22]. NSL is a typical colloidal self-assembly technique, which meets the effective nanofabrication in a highly parallel, wafer-scale, inexpensive way and uses hexagonal close-packed nanospheres of mainly polystyrene (PS) or silica as masks or templates for photolithography, evaporation, deposition, etching, imprinting, etc. [23, 24]. Because of the hexagonal close-packed arrangement of nanospheres, this results in a similar array of nanostructures. Moreover, such structures can exhibit the grating effects, for instance, the extraordinary optical transmission (EOT) performance of nanohole arrays, generally as a result of the excitation of surface plasmon polaritons (SPPs) [25]. This is specifically important for many possible applications such as surface-enhanced Raman scattering (SERS), enhanced detection of infrared (IR) vibrations, solar cells, and enhanced fluorescence [26–29].

In this work, our approach combines the advantages of the modified NSL, e.g., large-area and low-cost fabrication, with the conventional photolithographic technique to produce the desired metallic planar microlenses that are similar to the Odom’s “patches”. The realized microlenses as demonstrated can focus single wavelengths of light across the entire visible spectrum as well as the broadband white light with minimal divergence. Besides, via the simulation and experimental verification, the random defects commonly existing during the self-assembly procedure of nanospheres in nanohole arrays reveal no dramatic influences on the focusing performance of microlenses, which means the focal spots from different microlenses on the same wafer have the identical lateral dimensions, closing to the Rayleigh diffraction limit. The metallic nanohole-based microlenses and the so-developed NSL method presented here may open a door to design and fabricate a new type of microlenses for miniaturized transmissive planar micro-/nano-optical devices.

## Methods

Bottom-up self-assembly of dielectric PS nanospheres as a simple and low-cost route to form subwavelength nanoholes often suffers from severe defects, e.g., dislocations, multilayer, and point or area vacancies. To address these issues, we undertake experimental studies of the spin-coating parameters, including the spinning velocity, acceleration, suspension proportioning, and the hydrophilic modification of substrate surfaces, on the quality of the formed self-assembled arrays over the whole 4-in. glass wafer. Although the optimized parameters are adopted to reduce the major defects (vacancies and multilayer) and create the corresponding nanohole arrays through pattern transferring, some dislocations and vacancies are still inevitable and shifted to the final nanohole structures.

**Fig. 1:**
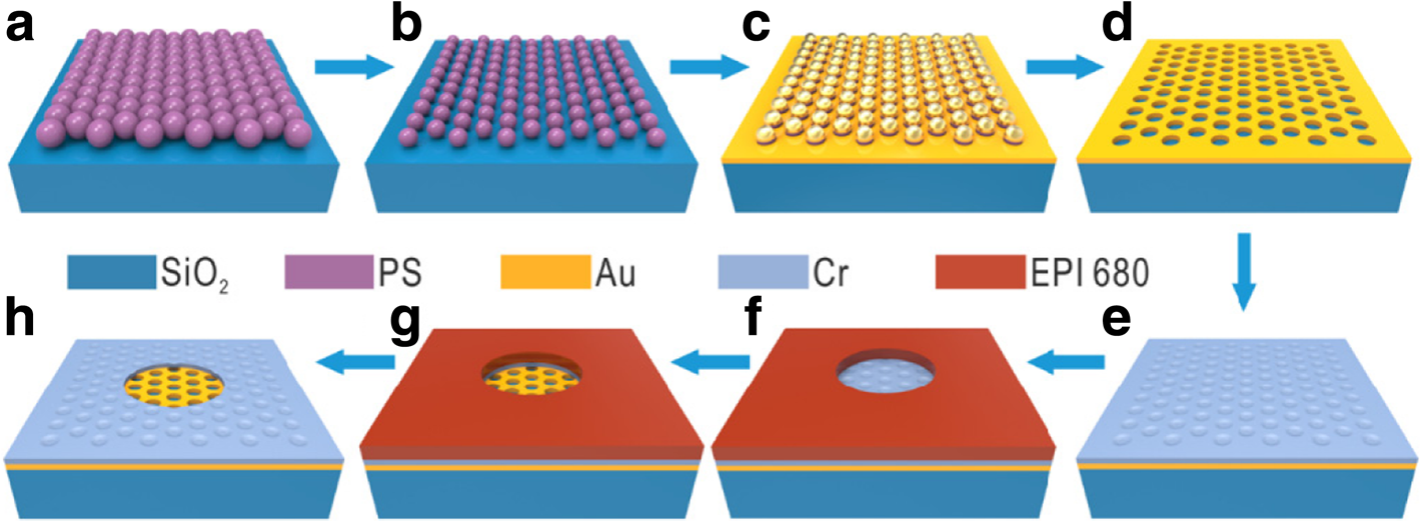
Schematic of the main process steps for fabricating the microlenses and their arrays. **a** Spin coating of monolayer PS nanospheres. **b** size shrinkage of PS nanospheres. **c** Au deposition. **d** removal of PS nanospheres. **e** Cr deposition. **f** transfer of microlenses patterns onto photoresist. **g** wet etching. **h** removal of photoresist

Figure 1 illustrates the combination of a bottom-up (spin-coating self-assembly of PS nanospheres) and a top-down technique (photolithography) for low-cost, parallel fabrication of microlenses and their arrays. Firstly, the PS nanospheres (from microParticles GmbH) are spin coated onto glass substrates, forming a monolayer mask of nanospheres with the hexagonal lattice (Fig. 1a). After deposition of nanospheres, their size is modified via the oxygen plasma in a parallel plate reactor (Plasma Reactor, 0.75 Pa, O_2_ 100 sccm, 80 W), as shown in Fig. 1b. In the next step, a 100-nm-thick gold layer is sputtered onto the monolayer PS nanospheres (Fig. 1c). After that, lift-off process is performed by the ultrasonic cleaning in tetrahydrofuran (THF), and a large-scale nanohole array is thus achieved (Fig. 1d). Then, the chromium (Cr) film is sputtered onto the first holey gold film (Fig. 1e) and patterned with the desired microlenses and their arrays by photolithography (Fig. 1f), which dominates the focusing performance of the ultimately achieved microlenses. Next, the Cr layer exposed by the opening areas is removed, leaving the holey gold nanoholes to transmit the incident light (Fig. 1g). After cleaning the residual photoresist, the designed microlenses and their arrays are realized (Fig. 1h).

**Fig. 2:**
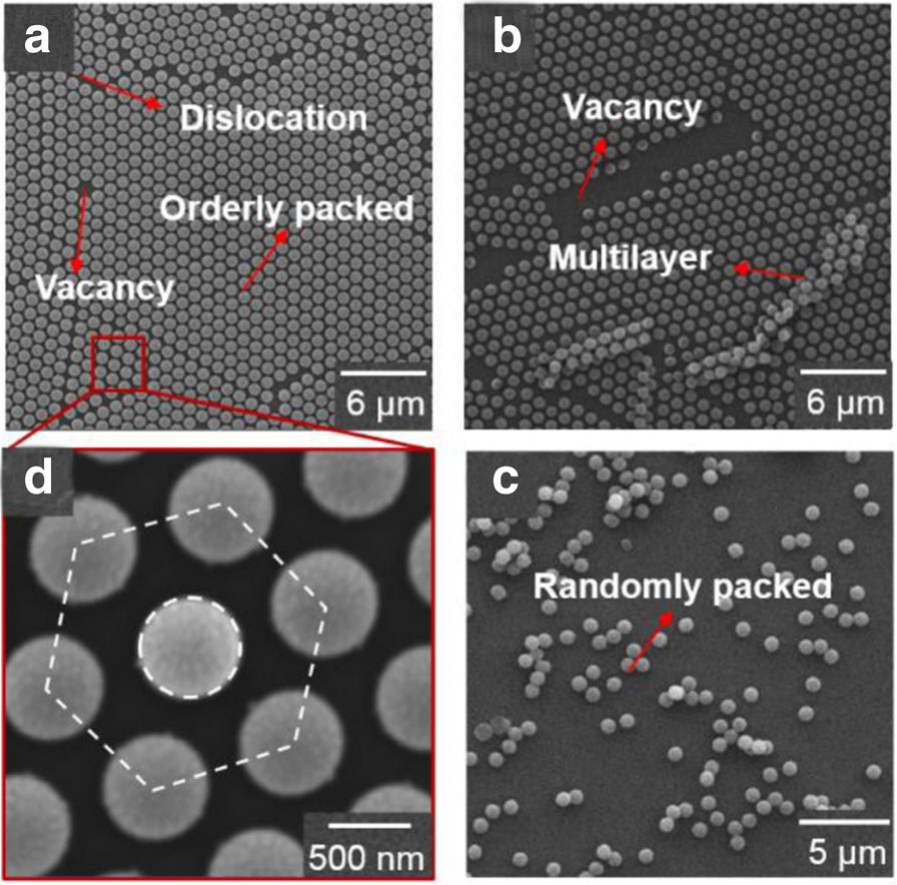
SEM images are shown for **a** self-assembled orderly packed monolayer PS nanospheres of 900 nm diameter, (**b**) PS size shrinkage by O_2_ plasma containing the defects of vacancies and multilayer, (**c**) randomly packed PS nanospheres, and (**d**) an enlarged view of a hexagonally packed PS unit

Figure 2 shows the representative scanning electron microscope (SEM) images displaying various self-assembled morphologies of PS nanospheres with the lattice spacing *P* = 900 nm, i.e., the diameter of PS nanospheres employed. The self-assembled monolayers of PS nanospheres are orderly packed in a hexagonal lattice on the glass substrates in Fig. 2a, d. However, dislocations that are shown as “cracks” are still present, due to electrostatic repulsion between the particles [30], as well as the point vacancies. Figure 2b, c illustrates area vacancies, multilayer, and randomly packed defects, which are distributed in certain regions with a poor controllability when the spin-coating parameters are not optimized or disturbed.

**Fig. 3:**
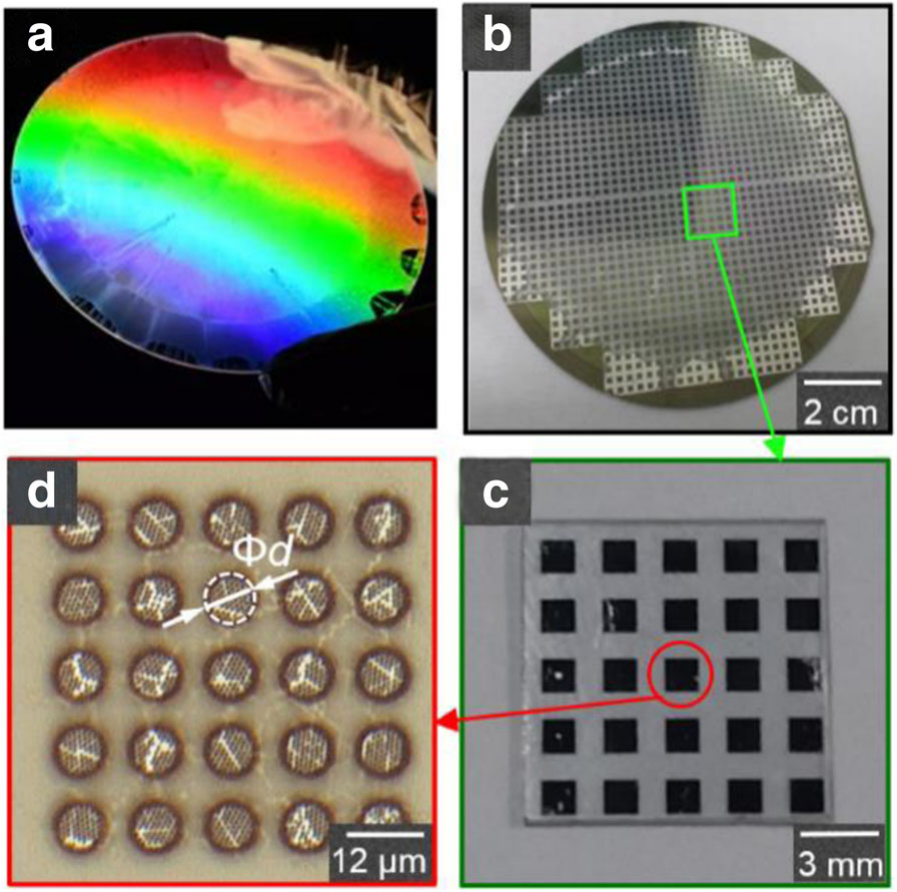
**a** Diffraction image of the fabricated 4-in. wafer-level monolayer nanosphere mask. Photographs of the fabricated microlenses and their arrays on a glass substrate in (**b**) the wafer level and (**c**) the chip level. **d** Optical microscope image of the 8-μm microlens and its 5 × 5 array spacing 4 μm apart

Figure 3 shows the result of the visible-light diffraction on the nanospheres mask and digital camera pictures of the 4-in. wafer and a 10 mm × 10 mm chip with various cells of microlenses. The individual microlens and its array are illustrated in Fig. 3d, in which the underlying nanoholes and the detached microlenses are clearly observed. It also reveals the existing random defects in the single microlenses.

**Fig. 4:**
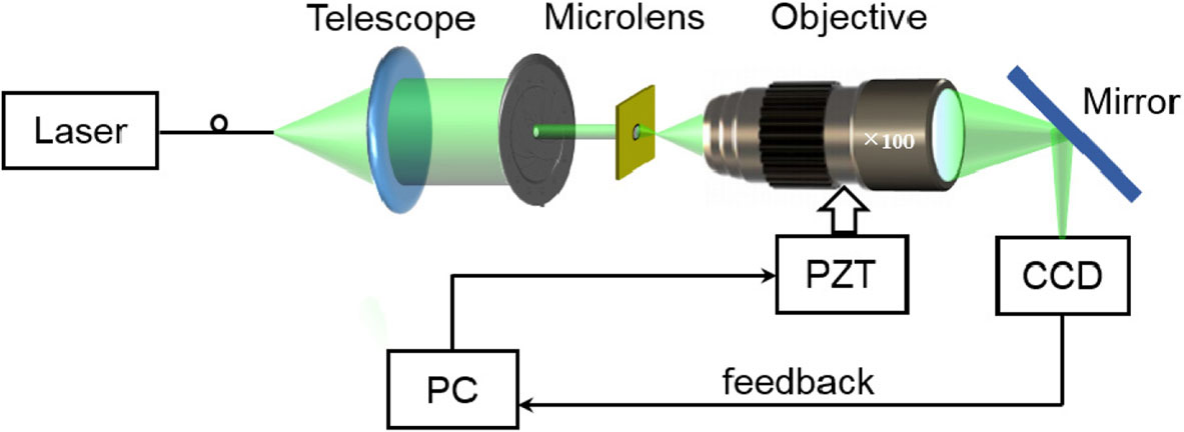
Experimental setup for characterizing the optical focusing performance of microlenses and their arrays. A 532-nm continuous-wave (CW) laser is expanded via a telescope in order to obtain a homogeneous beam. Passing through the microlens, the transmitted speckle pattern is collected by a microscope objective and measured by a CCD

In order to explore the focusing performance of the achieved microlenses, we compare the 3D finite-difference time-domain (FDTD) simulation results with the experimental testings. Our experimental setup, as described in Fig. 4, employing the Nikon inverted optical microscope as the main operating platform, is used to map the optical fields generated from the plane wave incident on the microlens. After transmitting through the microlens, a high-quality oil-immersion microscope objective (100×, NA = 1.49) images speckle patterns onto a CCD camera and is driven by the E-816 piezo controller (Physik Instrumente (PI)) with a stepping length of 100 nm. After collecting hundreds of 2D light slices, the 3D optical field along the propagating axis of microlens can be thus constructed.

## Results and Discussion

I. Focusing performance of microlenses

**Fig. 5:**
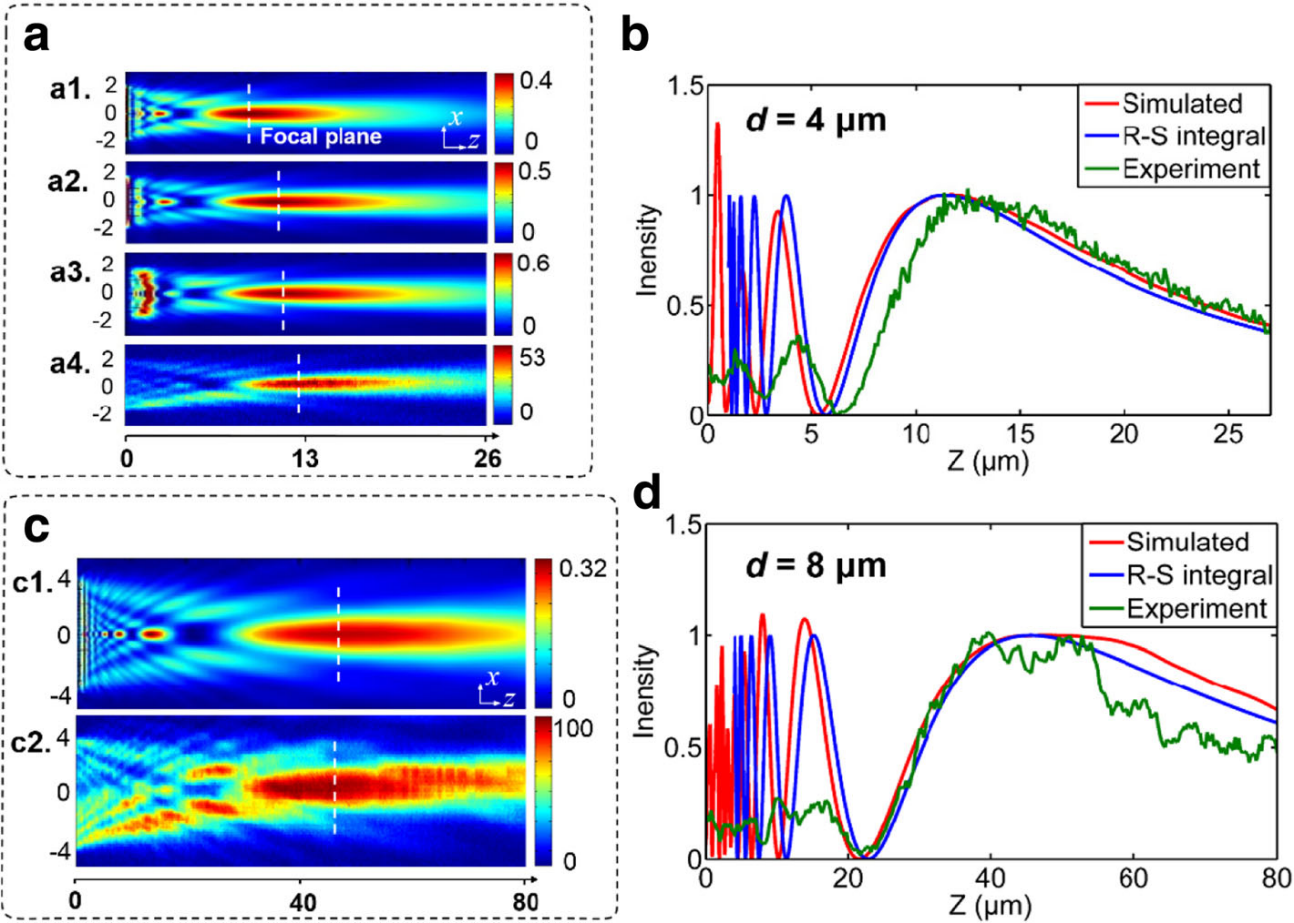
**a** Optical field mappings of the 4 μm microlens for the simulated cases of *a1 λ* = 633 nm, *P* = 522 nm; *a2 λ* = 532 nm, *P* = 522 nm; *a3 λ* = 532 nm, *P* = 900 nm; and *a4* the measured result of *λ* = 532 nm, *P* = 900 nm. **b** The axial light intensity of the calculated R-S integral, FDTD simulation, and optical measurement for the designed microlens of *d* = 4 μm. **c** The field intensity difference for the *c1* simulated and *c2* measured results when *d* = 8 μm at *λ* = 532 nm, *P* = 900 nm. **d** The axial intensity for the microlens of *d* = 8 μm. The intensity fields along the *y-z* planes are identically distributed as *x-z* planes

The 3D model of microlens with the diameter of *d* having circular nanoholes arranged in a hexagonal lattice is established by using the FDTD method. The collimated, *x*-polarized light with an operating wavelength of 532 nm is illuminated, a well-defined focal spot (location of maximum intensity) is observed in the *x*-*z* plane (the same as *y*-*z* plane since the electromagnetic field is distributed symmetrically) through the center of the 4-μm microlenses, and the full-width at half-maximum (FWHM) of the spot at the focal plane is 1.25 μm (Fig. 5a), which is close to the Rayleigh diffraction limit of 0.912 μm calculated by 0.61*λ*/NA [31]. Furthermore, the far-field optical patterns are simulated with the lattice spacings of 522 and 900 nm, and the operating wavelengths of 532 and 633 nm are selected. The simulated focal lengths are 12 and 10.4 μm for the 4-μm microlens at *λ* = 532 and 633 nm, respectively, and the value increases to 46 μm for the 8-μm microlens at *λ* = 532 nm, as shown in Fig. 5a, c. Because their focusing effects are not the consequence of the wavefront engineering, 4-μm microlenses with 522- or 900-nm lattice spacing have nearly identical focal spots, which validates that the focal length depends mainly on the lens size and the working wavelength.

The focal spot is subject to the classical Rayleigh diffraction limit because the far-field focusing does not originate from the recovery of evanescent field [32] or super-oscillations [33]. Hence, the dependence of focal length on the operating wavelength can be expressed by a relationship derived from the Rayleigh-Sommerfeld (R-S) integral [18]. From Fig. 5b, d, we can see that the calculated optical field distributions by the R-S integral agree very well with the FDTD simulation results for both cases. However, the measurement results show a slight difference due to the various errors introduced during the fabrication procedure and optical measurement. It is worth noting that for the 4-μm case, the measurement deviation in contrast to the simulation is 8.3%, compared to 1.1% for the 8-μm case. In other words, the microlenses with a larger diameter are more insensitive to the normal errors.

**Fig. 6:**
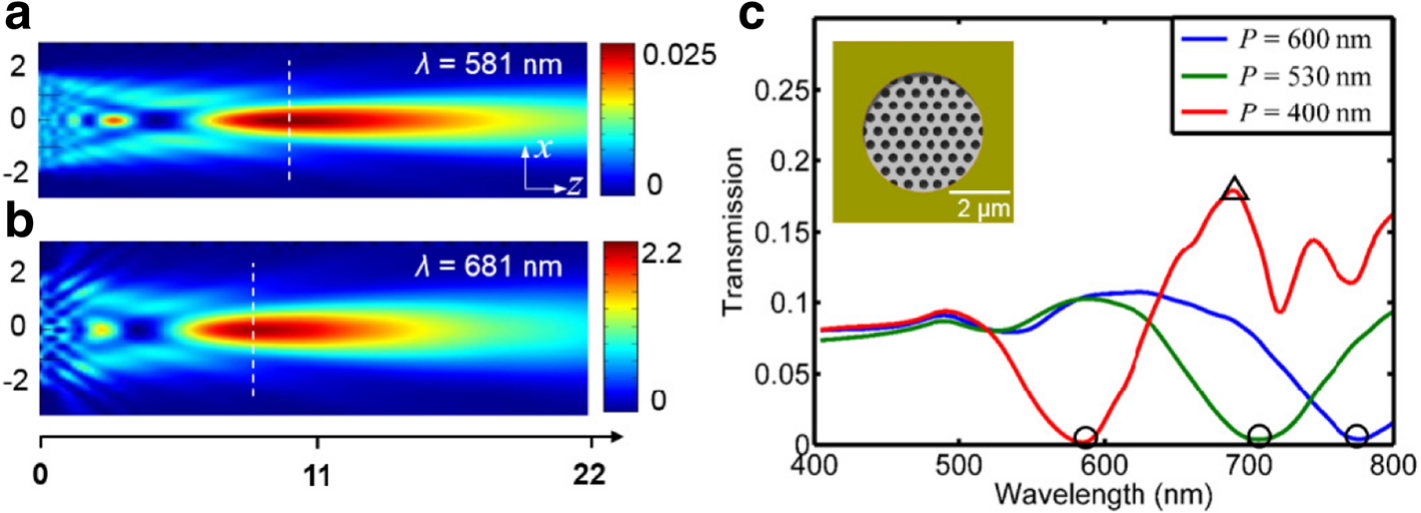
**a**, **b** Simulation results of the electric-field patterns of the 4-μm microlens when *P* = 400 nm at the working wavelength of *λ* = 581 nm (transmission dip) and 681 nm (peak), respectively. **c** Simulated total transmittance spectra for the microlenses corresponding to the lattice spacings *P* = 400, 530, and 600 nm in the frequency range of 400~800 nm, and the *inset* shows the model of a 4-μm microlens. The transmission dips and peak are flagged by *circles* and *triangle*, respectively

Since the focusing performance is irrelevant to the wavefront engineering, the optical throughput of the focal spot depends on the SP-enhanced transmission through the subwavelength apertures [18]. When the results from Fig. 6 are compared with the transmission spectra from the different microlenses, the enhanced transmissions and the suppressed transmissions are present at different wavelengths depending on the lattice spacing. According to previous reports [34], the selective spectral response was discovered to be stemming from the combined effect of the propagating surface plasmon resonance (PSPR) sustained at the metal/dielectric interface and the localized surface plasmon resonance (LSPR) around the nanoholes. As observed in Fig. 6c, the locations of the transmission dips, as implied by the circles, come up with a red shift along the *x*-coordinate axis as the lattice spacing increases, so it is with the transmission peaks. This endows the microlenses with unusual abilities to control the optical throughput at specific wavelengths and ensures microlenses being easily designed with a high-efficiency focusing. Figure 6a, b gives the field distributions of a 4-μm microlens for the case of *P* = 400 nm at the dip wavelength of 581 nm and the peak one of 681 nm, respectively. Except for a decrease in the focal length introduced by the increased wavelength, the intensity of the focal spot for the wavelength of 681 nm is almost 100 times more than that of *λ* = 581 nm.

II. Influences of random defects

Despite the fact that NSL is a highly parallel fabrication method to create large-area nanohole arrays in the microlenses and their arrays, one perceived problem of this technique is that the defects are randomly distributed throughout the nanohole layer of the microlenses. The defects are nearly inevitable during the self-assembly process of nanospheres, which are normally thought to fundamentally limit the resolution and penetration depth of the optical methods. However, it is astonishing that defects offer an unusual alternative to conventional periodic structures to manipulate light. Some random defects are demonstrated to improve, rather than deteriorate the sharpness of the focus in a specific optical experiment [35, 36]. Therefore, the influence of defects spawn from our fabrication process on the focusing performance of microlenses studied here is essential for practical applications and the further research about random photonic crystals.

**Fig. 7:**
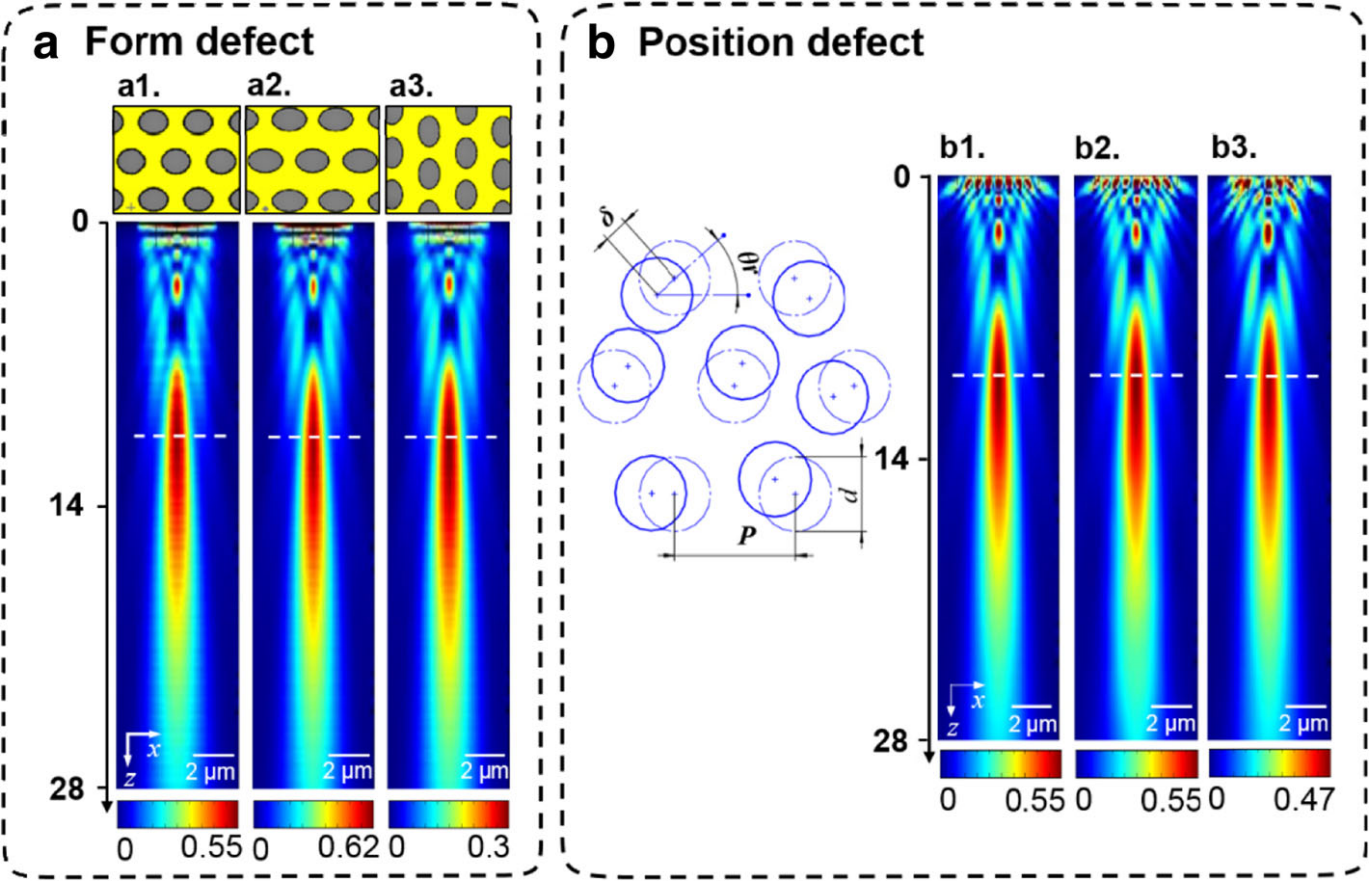
**a** Focal spots from microlenses are independent of the out-of-roundness error *σ* of nanoholes. The focusing properties do not show a clear change when *σ* = 0 (rounded nanoholes) in Fig. 5 is increased to *a1 σ* = 0.4, *a2 σ* = 0.7 with a horizontal distorted direction, and *a3 σ* = 0.7 with a perpendicular distorted direction. **b** Introduction of spatial randomness into the positions of nanoholes. Deviation directions are randomly different from hole to hole, but the deviation length *δ* is kept constant for each hole. The same focusing patterns are obtained when the deviation length *b1 δ* = 0, *b2 δ* = 50 nm, and *b3 δ* = 100 nm

Apart from the abovementioned vacancies, dislocations, and multilayer defects that are generated from the self-assembly procedure of nanospheres, the shape deformation of nanoholes may also exist in the ultimate microlenses during the PS shrinkage and PS removal as a result of the imbalanced O_2_ plasma etching. Therefore, these defects we considered can be classified as the form and position defects. To demonstrate the impact of the form defects on the focusing performance of microlenses, we present the microlenses with different out-of-roundness *σ* in the nanoholes when their common fill factor is 0.33 and the corresponding optical focusing images are given in Fig. 7a. Obviously, these focusing patterns for cases of *σ* = 0.4 and *σ* = 0.7 are almost the same except the slight variation of foci intensity. More obviously, as seen in Fig. 7a, the similar foci patterns in a1, a2, and a3 indicate that the increased degree of deformation and the change of deformation direction impose negligible influence on the focusing properties of microlenses.

To explore the influences of position defects, we deviate the positions of nanoholes to different directions with a length *δ*. The deviation direction of each hole is randomly distributed from hole to hole and kept constant for each *δ* (see Fig. 7b). With the increase of *δ*, the nanoholes deviate from the perfectly close-packed state and become “more random.” Three similar focusing patterns of microlenses regarding different random positions of nanoholes, *δ* = 0, 50, and 100 nm, are obtained. Furthermore, it is observed that a slight decrease in foci intensity appears on the field profile with a more random nanohole array. Above all, it reveals that the form and position defects within microlenses play little effect on the focusing performance and mostly just modulate the foci intensity.

III. Focusing performance of microlens arrays

**Fig. 8:**
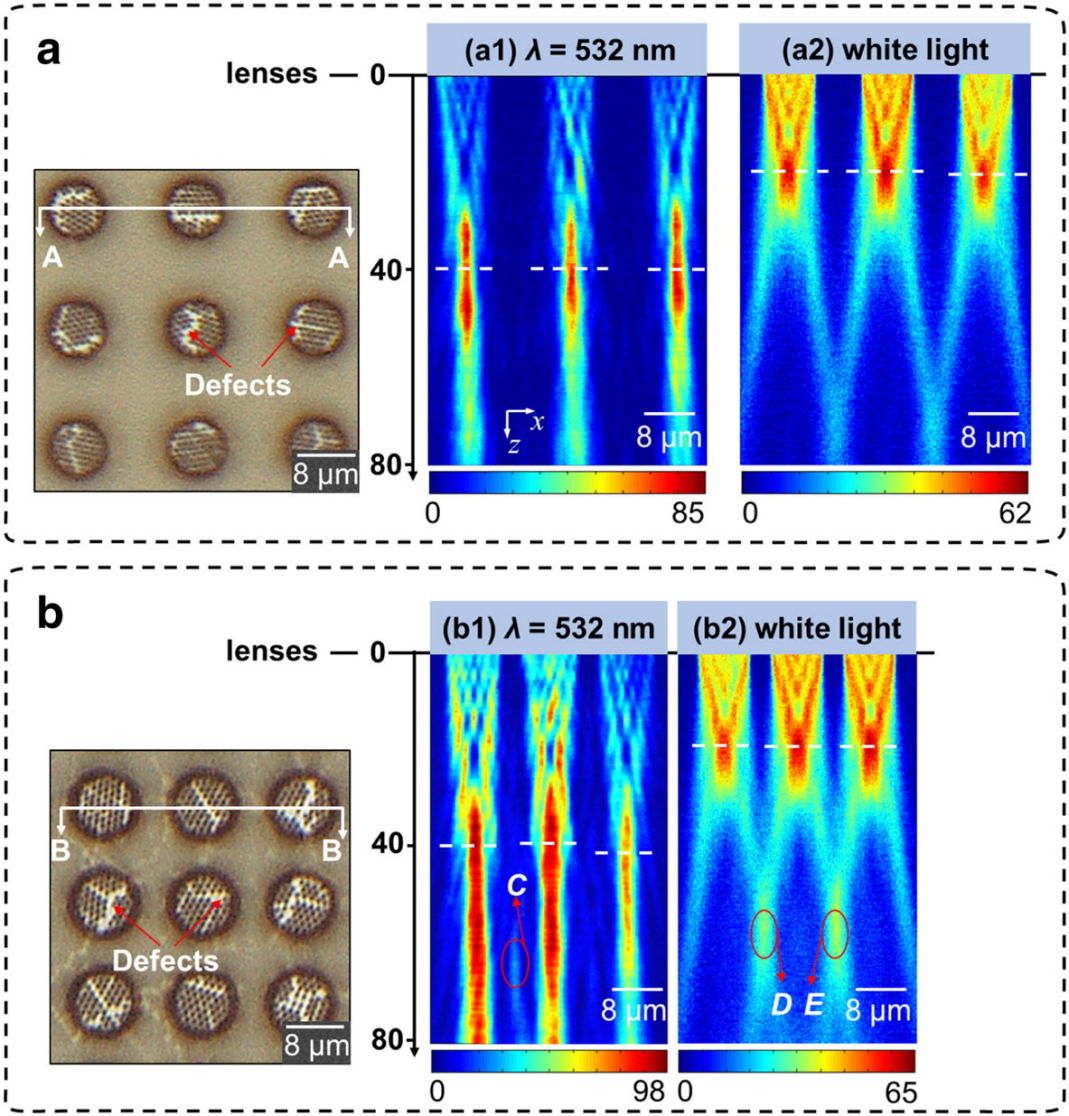
**a** Optical microscope images for the fabricated 3 × 3 microlens array spacing 8 μm apart when *d* = 8 μm and the measured optical patterns (*A*–*A*) under *(a1)* the single wavelength of *λ* = 532 nm and *(a2)* the broadband illumination. Identical foci from individual microlenses are observed. **b** Experimental results (*B*–*B*) for the 3 × 3 microlens array spacing 4 μm under *(b1)* the single wavelength of *λ* = 532 nm and *(b2)* the broadband illumination. The coupling effect between two adjacent microlenses, as denoted by the regions *C*, *D*, and *E*, can be observed

Figure 8 shows the fabricated 3 × 3 microlenses array with different spacings and the experimentally measured optical patterns under *λ* = 532 nm, as well as the broadband illumination. Note that the focal spots from microlenses with more dislocations in the array are weaker than those from other microlenses in Fig. 8b. It is because the dislocation defects effectively reduce the number of nanoholes contributing to the optical interference pattern. Further, the results show excellent agreement with those obtained by the FDTD simulations that the defects mainly affect the foci intensity. In addition, the microlenses can focus the broadband white light (Fig. 8 (a2), and (b2)) due to the minimal chromatic aberration. The focal spots under the white light illumination have the similar lateral dimensions as those under a single wavelength, while the broadband focal length is approximately the average of the focal lengths at the SP-enhanced wavelengths. In addition, the focusing coupling effect in microlens array which we had analyzed in our previous research [37] emerges in the obtained focusing patterns as the regions C, D, and E flagged in Fig. 8 (b1) and (b2).

## Conclusions

To sum up, we have demonstrated for the first time that the NSL technique as a highly parallel and low-cost method can be used to fabricate the metallic planar microlenses functioning over the entire visible spectrum. Supported by the simulated and experimental results, the focusing properties of microlenses can be explained by a combination of both optical interference and surface plasmon effects. Taking into consideration the lattice spacing and diameter of nanoholes, the microlenses can be tailored to provide high transmission at specific wavelengths. The focusing performance of microlenses from the perfect to the defective state is exploited by the FDTD method. Both the simulations and experiments clarify that the random defects in nanohole arrays simply affect the focusing efficiency of microlenses and the focusing coupling effect as predicted occurs under both the single wavelength and broadband illumination. The broadband focusing capability, miniaturized size, and versatile fabrication technique all together open a great potential for compact and inexpensive all-optical or opto-electronic devices such as photovoltaics [26], color filters [38], and refractive index sensing [39].

## Abbreviations

3D: Three-dimensional
CCD: Charge-coupled device
FDTD: Finite-difference time-domain
FWHM: Full-width at half-maximum
LIL: Laser interference lithography
NA: Numerical aperture
NSL: Nanosphere lithography
PS: Polystyrene
SEM: Scanning electron microscope
